# Elevated TIMP-1 expression is associated with a prometastatic phenotype, disease relapse, and poor survival in neuroblastoma

**DOI:** 10.18632/oncotarget.19664

**Published:** 2017-07-28

**Authors:** Pritha Paul, Eric J. Rellinger, Jingbo Qiao, Sora Lee, Natasha Volny, Chandrasekhar Padmanabhan, Carmelle V. Romain, Bret Mobley, Hernan Correa, Dai H. Chung

**Affiliations:** ^1^ Section of Surgical Sciences, Department of Surgery, Vanderbilt University Medical Center, Nashville, TN 37232, USA; ^2^ Department of Cancer Biology, Vanderbilt University Medical Center, Nashville, TN 37232, USA; ^3^ Department of Pathology, Vanderbilt University Medical Center, Nashville, TN 37232, USA; ^4^ Department of Pediatric Surgery, Vanderbilt University Medical Center, Nashville, TN 37232, USA

**Keywords:** TIMP-1, neuroblastoma, metastasis, liver, LM2

## Abstract

Approximately two-thirds of patients with neuroblastoma are found to have metastatic disease at time of diagnosis with frequent skeletal, lymph node, central nervous system, and liver involvement. Using a serial *in vivo* splenic injection model, we have isolated an aggressive subclone (BE(2)-C/LM2) from *MYCN*-amplified neuroblastomas that demonstrate an enhanced propensity to develop metastatic liver lesions. BE(2)-C/LM2 subclone cells demonstrate increased adherent, soft agar colony and tumorsphere growth *in vitro*. Components of the tumor microenvironment regulate cancer progression, via networks of cytokines and growth factors. Cytokine array analysis identified increased TIMP-1 in the plasma of mice injected with BE(2)-C/LM2 subclone cells, leading us to hypothesize that TIMP-1 may play a role in our observed prometastatic phenotype. Immunoblotting and ELISA demonstrated enhanced endogenous TIMP-1 expression in our isolated neuroblastoma subclone. Silencing endogenous TIMP-1 successfully blocked *in vitro* proliferation, soft agar colony formation and tumorsphere formation by BE(2)-C/LM2 cells. Stable RNA interference of endogenous TIMP-1 failed to reverse the prometastatic phenotype of our BE(2)-C/LM2 subclone in our liver metastasis model, suggesting that endogenous TIMP-1 levels may not be an essential component of this *in vivo* behavior. Notably, tissue microarray analysis and Kaplan-Meier by gene expression demonstrates that elevated TIMP-1 expression is correlated with increased disease relapse and mortality in patients with neuroblastoma. Taken together, our study identifies TIMP-1 as a novel soluble factor that is associated with a prometastatic phenotype in our *in vivo* model and adverse outcomes in patients with neuroblastoma.

## INTRODUCTION

Neuroblastoma is the most common extra-cranial solid tumor in children, originating from the neural-crest elements of the sympathoadrenal axis [1]. Despite advances in multi-modality therapy, survival rates for advanced-stage disease remain poor at 30-50% for children with high-risk disease [2]. Of those children with high-risk neuroblastoma who achieve remission with induction therapy, half will relapse, most commonly with evidence of distant metastasis to the skeleton, bone marrow, central nervous system, or liver. Significant contributions have been made in the identification of critical drivers of primary tumor formation, such as *MYCN* amplification and *ALK* mutations, but few studies have been devoted to the identification of consistent features in neuroblastoma metastasis.

Tumor-host microenvironmental factors are being increasingly recognized for their roles in mediating tumor progression and metastasis. The release of soluble growth factors and cytokines has been shown to facilitate angiogenesis, tumor invasion, and potentially provide local immunosuppressive effects to mediate distant tumor metastasis [3, 4]. In neuroblastoma, vascular endothelial growth factor and interleukin-6 are two cytokines that have been shown to promote tumor growth and metastasis [5]. Identifying growth factors/cytokines that mediate tumor progression/metastasis and can be readily measured in plasma has the potential to: 1) identify targetable pathways for therapeutic intervention of high-risk tumor, 2) risk stratify patients at time of initial diagnosis into more aggressive or conservative treatment strategies, and 3) surveil patients for evidence of disease recurrence.

Of children with neuroblastoma presenting with metastatic disease, 70% of metastases occur in the bone marrow, 31% have lymph node involvement, and 30% present with liver metastases [6]. At present, no *in vivo* models have been generated that faithfully recapitulate the metastatic pattern of neuroblastoma. A recent study by Seong et al. [7] utilized repeated intracardiac injection to identify *in vivo* selected cells that give rise to bone marrow and central nervous system metastases, identifying CADM1, SPHK1, and YAP/TAZ as critical mediators of bone marrow and central nervous system metastasis. However, their intracardiac injection model failed to consistently yield metastases to the liver.

Herein, we describe an *in vivo* splenic injection model that gives rise to liver metastases. *In vivo* selected cells (BE(2)-C/LM2) in our model give rise to liver metastases with 100% frequency, decreased metastatic latency, and decreased overall survival compared to parental cells. We performed cytokine array analysis using plasma from mice injected with BE(2)-C/Luc or BE(2)-C/LM2 and report increased tissue inhibitor of metalloproteinases (TIMP)-1 expression in mice injected with BE(2)-C/LM2 cells. TIMPs are a family of cytokines (TIMP-1, -2, -3, and -4) that are known as endogenous inhibitors of matrix metalloproteinases and associated with advanced disease in numerous adult cancers. Interestingly, targeted down-regulation of TIMP-1 (siTIMP-1 or shTIMP-1) using interfering RNA in neuroblastoma decreased *in vitro* proliferation in adherent, soft agar, and neurosphere conditions, but failed to impede the prometastatic LM2 phenotype in our liver metastasis model. Utilizing publically-available datasets, we have demonstrated that high TIMP-1 gene expression is significantly associated with disease relapse and mortality in patients with neuroblastoma. Taken together, these findings suggest that TIMP-1 may play a role in advanced stage neuroblastoma progression.

## RESULTS

### *In vivo* selected cells (BE(2)-C/LM2) have enhanced metastatic capacity to the liver

**Figure 1:**
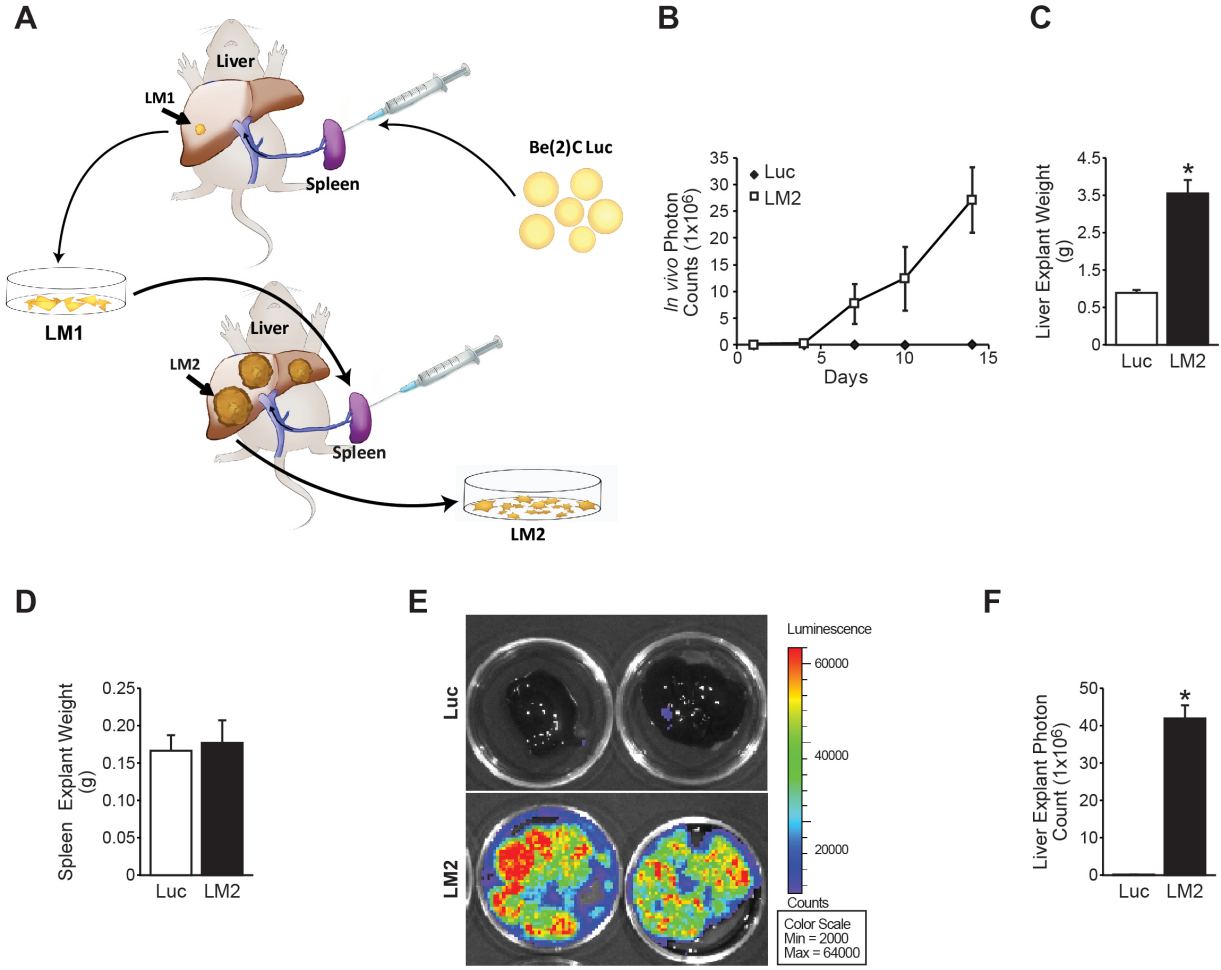
Serial splenic injection isolates a prometastatic subclone of neuroblastoma (BE(2)-C/LM2) **(A)** Schematic representation of the *in vivo* selection model used to isolate aggressive sub-clones of neuroblastoma cells from murine liver metastatic foci. **(B)** Serial *in vivo* bioluminescent imaging demonstrates enhanced metastatic burden in mice injected with the BE(2)-C/LM2 cells as compared to parental BE(2)-C/Luc cells. **(C)** BE(2)-C/LM2 liver explant weights were significantly greater than livers isolated from mice injected with BE(2)-C/Luc cells. **(D)** No differences in spleen weights were noted between groups (p=0.76). **(E, F)** Explant bioluminescence demonstrates enhanced neuroblastoma tumor burden in the liver of mice injected with BE(2)-C/LM2 cells (mean ± SEM; *=*p*<0.001).

In this study, we proposed that serial *in vivo* selection in our splenic injection model would isolate a subclone of neuroblastoma capable of eliciting enhanced liver metastasis burden. Human neuroblastoma BE(2)-C cells expressing luciferase [BE(2)-C/Luc] were injected into the spleen of male, athymic nude mice (Figure 1A). After four weeks, metastatic liver lesions were observed by *in vivo* bioluminescence, harvested, and grown *in vitro* using zeocin as a selection marker for cells expressing luciferase. Using these cells, intrasplenic injection was repeated and subsequent liver metastatic foci were isolated and named BE(2)-C/LM2. Figure 1A provides a schematic representation of the *in vivo* selection model used to isolate prometastatic subclone of neuroblastoma cells from murine liver lesions.

Upon reimplantation of the BE(2)-C/LM2 subclone, we observed decreased liver metastasis latency and abrupt onset of overwhelming disease burden within two weeks of implantation. To quantify this observation, BE(2)-C/Luc or BE(2)-C/LM2 cells were evaluated head-to-head in our liver metastasis model. Two weeks following splenic injection, mice injected with BE(2)-C/LM2 developed overwhelming liver metastasis associated with abdominal distension and hemoperitoneum. *In vivo* bioluminescence imaging demonstrated a significant difference in bioluminescence consistent with the increased intraabdominal tumor burden (Figure 1B). Mice were subsequently sacrificed, and the liver and spleen were isolated, weighed, and bioluminescent imaging was performed to quantify splenic primary and liver metastatic tumor burden. Mice injected with BE(2)-C/LM2 cells demonstrated significant hepatomegaly with increased liver explant weight (Figure 1C; 3.655g vs. 1.258g; *p*<0.001). Notably, no differences in spleen weight (Figure 1D) or bioluminescence were noted, suggesting that the primary injection site formed tumors of comparable size. Elevated liver tumor burden with BE(2)-C/LM2 injection was confirmed by liver explant bioluminescence imaging (Figure 1E and 1F).

To discern whether BE(2)-C/LM2 were protumorigenic regardless of implantation site, we performed a subcutaneous xenograft trial comparing BE(2)-C/Luc and BE(2)-C/LM2 in the bilateral flanks of athymic nude mice. No differences in the rate of subcutaneous tumor formation (4/5 vs. 4/5), serial tumor volume measurement (p=0.73), or subcutaneous tumor explant mass were noted (Supplementary Figure 1; 0.428g vs. 0.408g; *p*=0.94). Taken together, these results validated that our *in vivo* selection model isolated a neuroblastoma subclone capable of enhanced liver metastasis in our murine model system.

### BE(2)-C/LM2 subclone demonstrated enhanced *in vitro* growth

**Figure 2:**
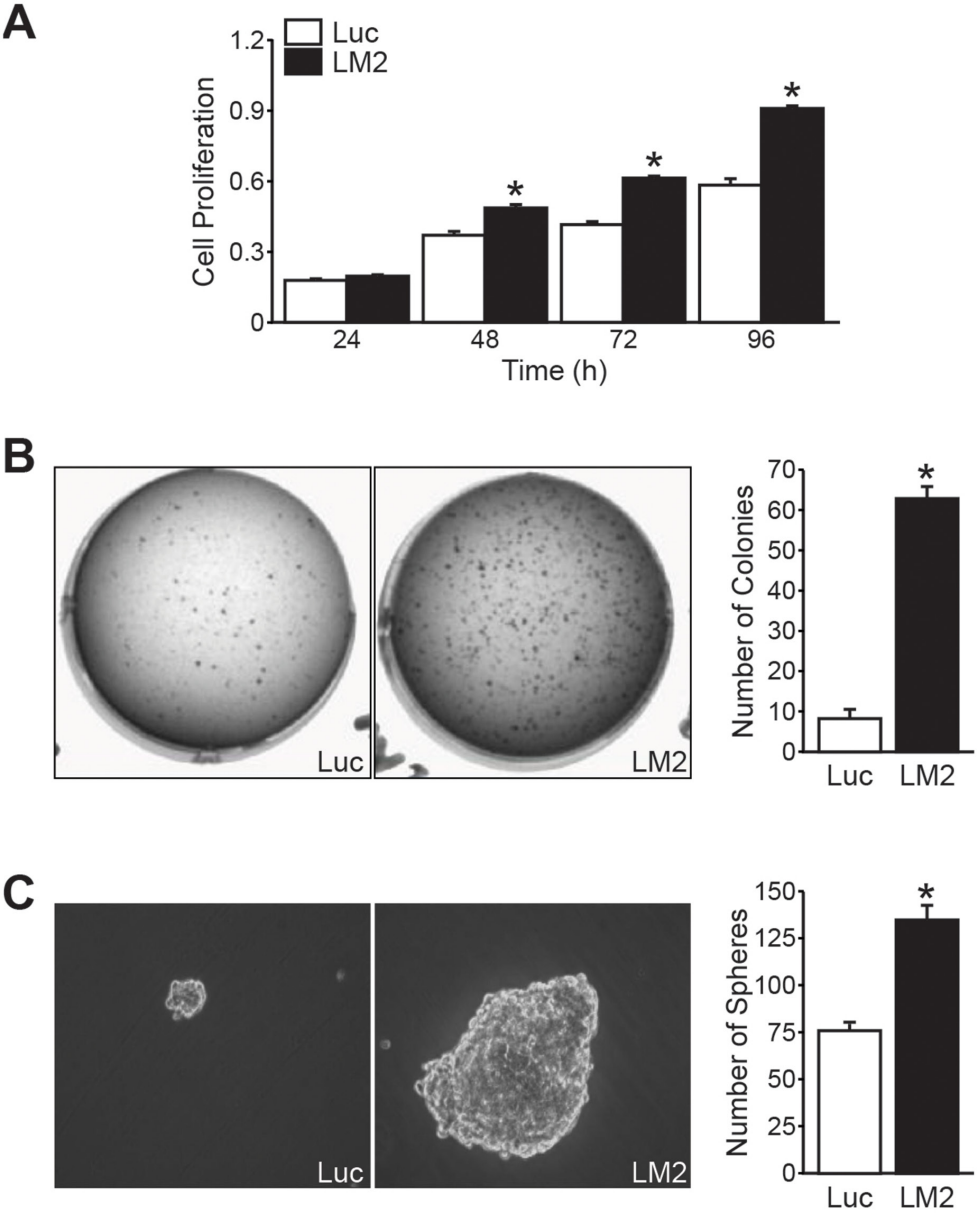
BE(2)-C/LM2 demonstrates enhanced growth potential *in vitro* **(A)** BE(2)-C/LM2 cells have enhanced proliferative rates in our tetrazolium-based assay compared to parental control (BE(2)-C/Luc) starting at 48 h. **(B)** Enhanced anchorage-independent growth and **(C)** neurosphere formation was also demonstrated in the BE(2)-C/LM2 subclone (mean ± SEM; *= *p*< 0.05).

We next sought to characterize the growth potential of the BE(2)-C/LM2 subclone *in vitro* by conducting adherent, soft agar, and neurosphere growth assay as surrogates of proliferation, transformation, and isolation of stem cell-like populations, respectively [8, 9]. BE(2)-C/LM2 cells demonstrated enhanced proliferation in our tetrazolium-based assays (Figure 2A), soft agar colony conditions (Figure 2B), and neurosphere conditions in comparison to their parental control (Figure 2C). These findings demonstrate that our *in vivo* selected subclone demonstrates enhanced growth capacity *in vitro.*

### Cytokine profiling in mice injected with *in vivo* selected cells

**Figure 3:**
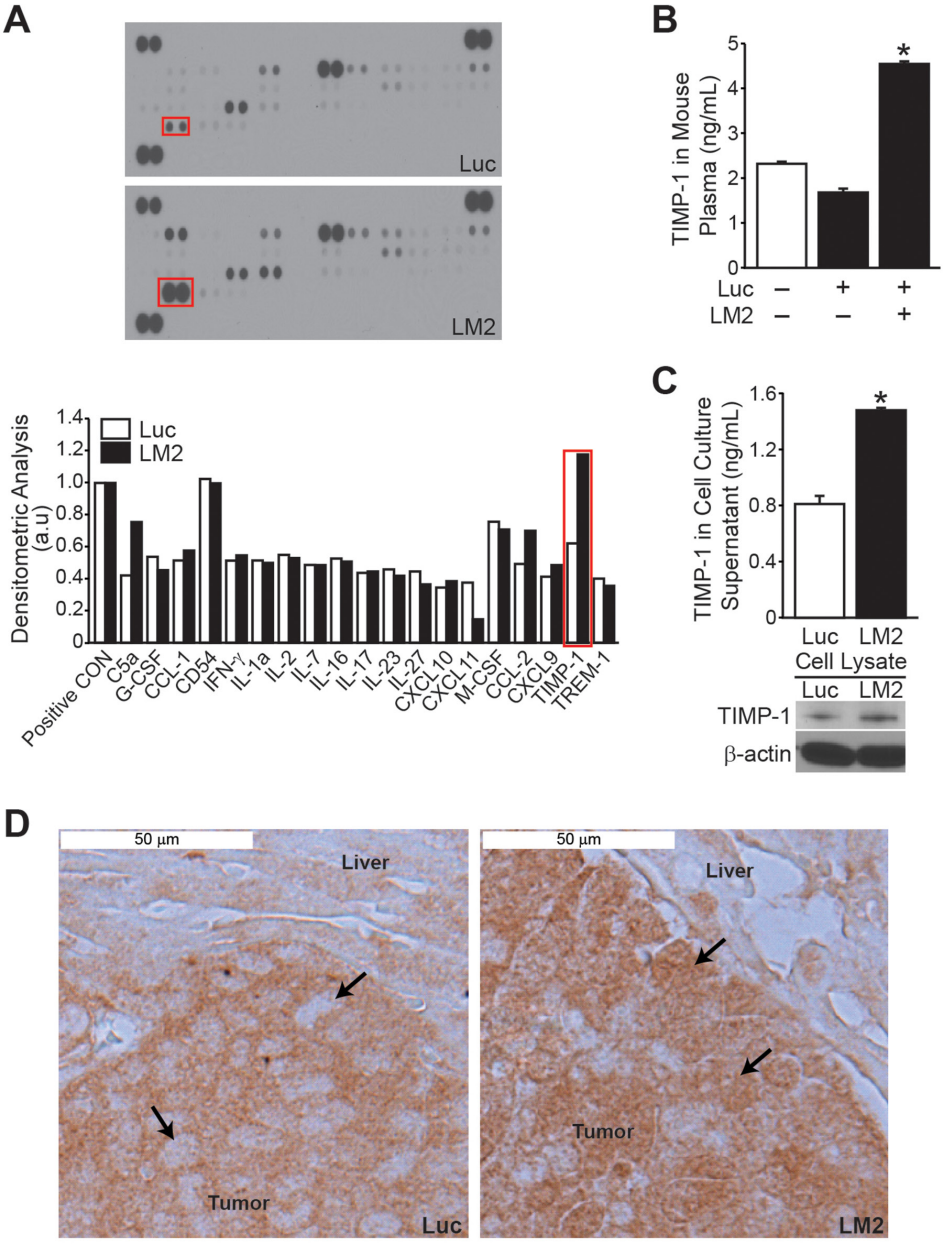
TIMP-1 expression is increased in BE(2)-C/LM2 subclone and host plasma Cytokine array analysis using murine plasma showed higher TIMP-1 levels in mice injected with BE(2)-C/LM2 cells when compared to BE(2)-C/Luc controls. **(B)** Increased plasma TIMP-1 expression in mice injected with BE(2)-C/LM2 cells was confirmed by murine TIMP-1-specific ELISA. **(C)** Endogenous tumor TIMP-1 secretion and expression was higher in BE(2)-C/LM2 cells in comparison to BE(2)-C/Luc parental cells as assessed by human TIMP-1-specific ELISA and immunoblotting, respectively. **(D)** Immunohistochemical analysis indicated a differential expression of TIMP-1 in the hepatic lesions of mice injected with BE(2)-C/Luc versus BE(2)-C/LM2 (arrows indicate tumor cells within the hepatic micrometastases; mean ± SEM; * =*p*< 0.05).

To identify differential expression of cytokines that may be responsible for the aggressive nature of the BE(2)-C/LM2 subclone, we utilized a mouse-specific cytokine array. Differential expression of C5a, CXCL11, CCL-2 and TIMP-1 was observed (Figure 3A). For the remainder of the study, we focused on TIMP-1 as the fold change increase for this cytokine was greater than that observed for C5a, CXCL11 or CCL-2. Moreover TIMP-1 has a described role as a biomarker in numerous adult solid tumors [10-14]. As such, we hypothesized that TIMP-1 may be a critical regulator of metastasis in neuroblastomas. We first validated that murine secretion of TIMP-1 was increased by performing mouse-specific ELISA and demonstrated an approximately 3-fold increase in comparison to mice injected with BE(2)-C/Luc cells (Figure 3B). Interestingly, similar to murine plasma, basal secretion and expression of endogenous TIMP-1 was higher in BE(2)-C/LM2 cells in comparison to BE(2)-C/Luc parental cells as assessed by human-specific TIMP-1 ELISA and immunoblotting, respectively (Figure 3C). The specificity of mouse- and human-specific TIMP-1 ELISA was confirmed (Supplementary Figure 2A).

Immunohistochemical analysis of liver explants was also performed to visualize the expression of TIMP-1 within our *in vivo* model. The hepatic lesions of BE(2)-C/Luc cells demonstrated TIMP-1 expression restricted to the cytoplasmic compartment, while a more global expression of TIMP-1 was observed in hepatic lesions from mice injected with BE(2)-C/LM2 (Figure 3D). Additional tumor explant sections are shown in Supplementary 2B with dual H&E and TIMP-1 staining for ease of distinguishing tumor deposits within the liver bed. Overall our observations demonstrate increased expression of host- and tumor-derived TIMP-1 in our prometastatic model, suggesting that TIMP-1 may play an important role in mediating the aggressive behavior seen in the subclone.

### Silencing TIMP-1 inhibited neuroblastoma growth *in vitro*

**Figure 4:**
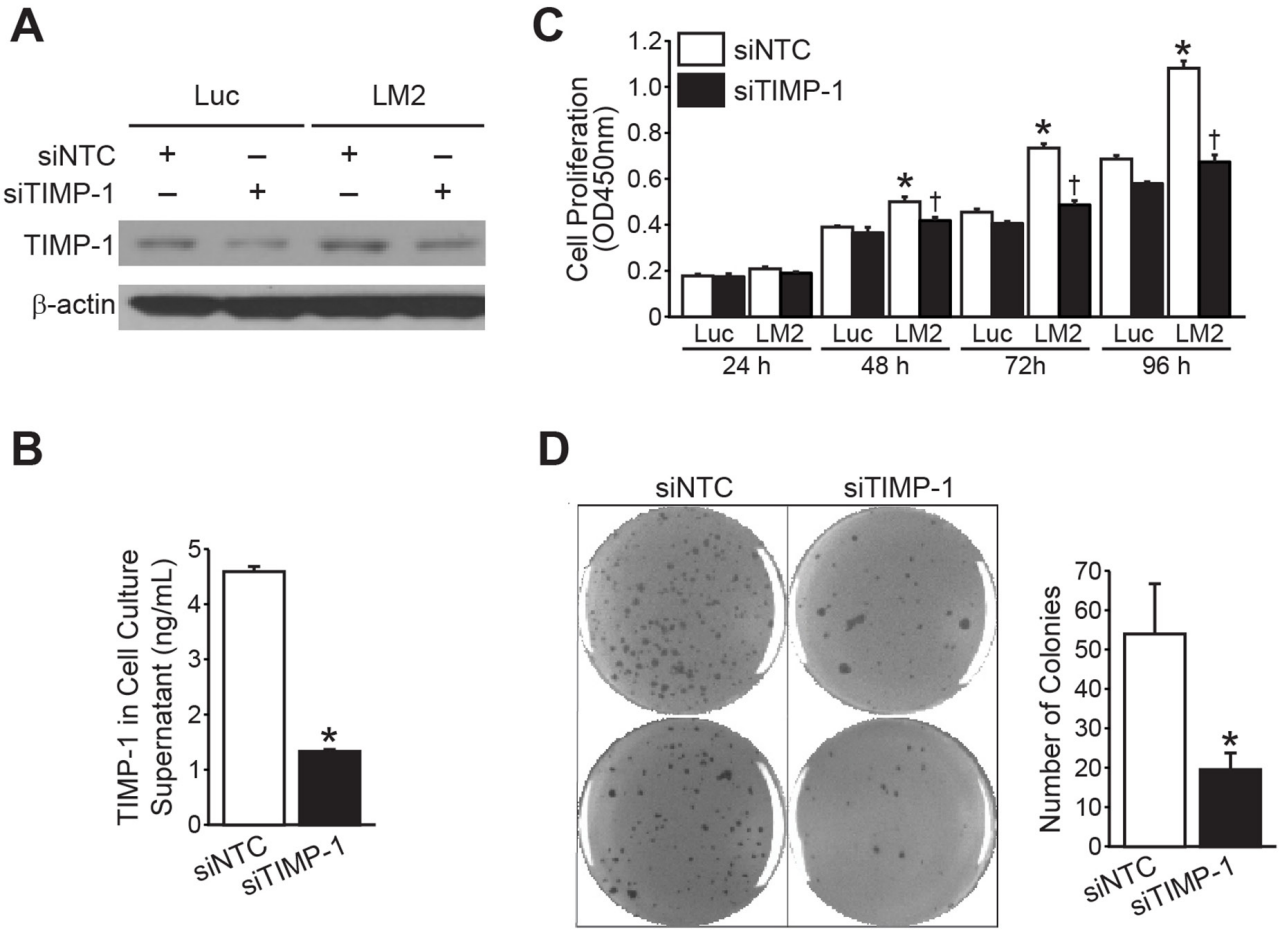
RNA interference of TIMP-1 blocks BE(2)-C/LM2 growth *in vitro* **(A, B)** TIMP-1 silencing by siRNA was confirmed by immunoblotting and human-specific TIMP-1 ELISA, respectively. **(C)** Silencing TIMP-1 decreased the proliferation rate of BE(2)-C/LM2 cells but not that of BE(2)-C/Luc cells. **(D)** Targeting TIMP-1 using siRNA inhibited anchorage-independent growth of BE(2)-C/LM2/siTIMP-1 cells in comparison to BE(2)-C/LM2/siNTC cells (mean ± SEM; *LM2 vs. Luc =*p*< 0.05; † LM2siNTC vs. LM2 siTIMP-1 = p<0.05).

To assess whether endogenous expression of TIMP-1 provided a growth advantage to neuroblastoma cells, we transiently transfected BE(2)-C/Luc and BE(2)-C/LM2 cells with siRNA against either TIMP-1 (siTIMP-1) or non-targeting control (siNTC). TIMP-1 silencing was confirmed by immunoblotting and human-specific TIMP-1 ELISA (Figure 4A and 4B, respectively). Cell proliferation was analyzed over a 96h time course. Silencing TIMP-1 in BE(2)-C/Luc demonstrated no significant difference in the proliferative capacity, but inhibited the proliferation of BE(2)-C/LM2 to a rate comparable to the parental BE(2)-C/Luc cells (Figure 4C). Furthermore, targeting TIMP-1 using siRNA significantly inhibited anchorage-independent growth in BE(2)-C/LM2/siTIMP-1 cells in comparison to BE(2)-C/LM2/siNTC cells (Figure 4D).

To confirm the specificity of target silencing, we also made use of shRNA-mediated TIMP-1 silencing. For this, BE(2)-C/LM2 cells were stably-transfected with shRNA against control (shCON) or TIMP-1 (shTIMP-1). Western blotting and ELISA validated our knockdown in BE(2)-C/LM2 cells (Supplementary Figure 3A and 3B). Similar to siRNA-mediated TIMP-1 silencing, the rate of cell proliferation was markedly reduced in BE(2)-C/LM2/shTIMP-1 in comparison to BE(2)-C/LM2/shCON (Supplementary Figure 3C). Anchorage-independent growth was similarly decreased in BE(2)-C/LM2/shTIMP-1 cells, as compared to BE(2)-C/LM2/shCON (Supplementary Figure 3D). These findings confirmed the anti-proliferative effect of TIMP-1 silencing *in vitro* as observed with siRNA-mediated silencing (Figure 4C and 4D). Hence, down-regulating the expression of TIMP-1 using either siRNA or shRNA, appears to decrease the growth potential of the aggressive LM2 subclone *in vitro.*

### Silencing endogenous TIMP-1 fails to rescue the prometastatic LM2 phenotype

**Figure 5:**
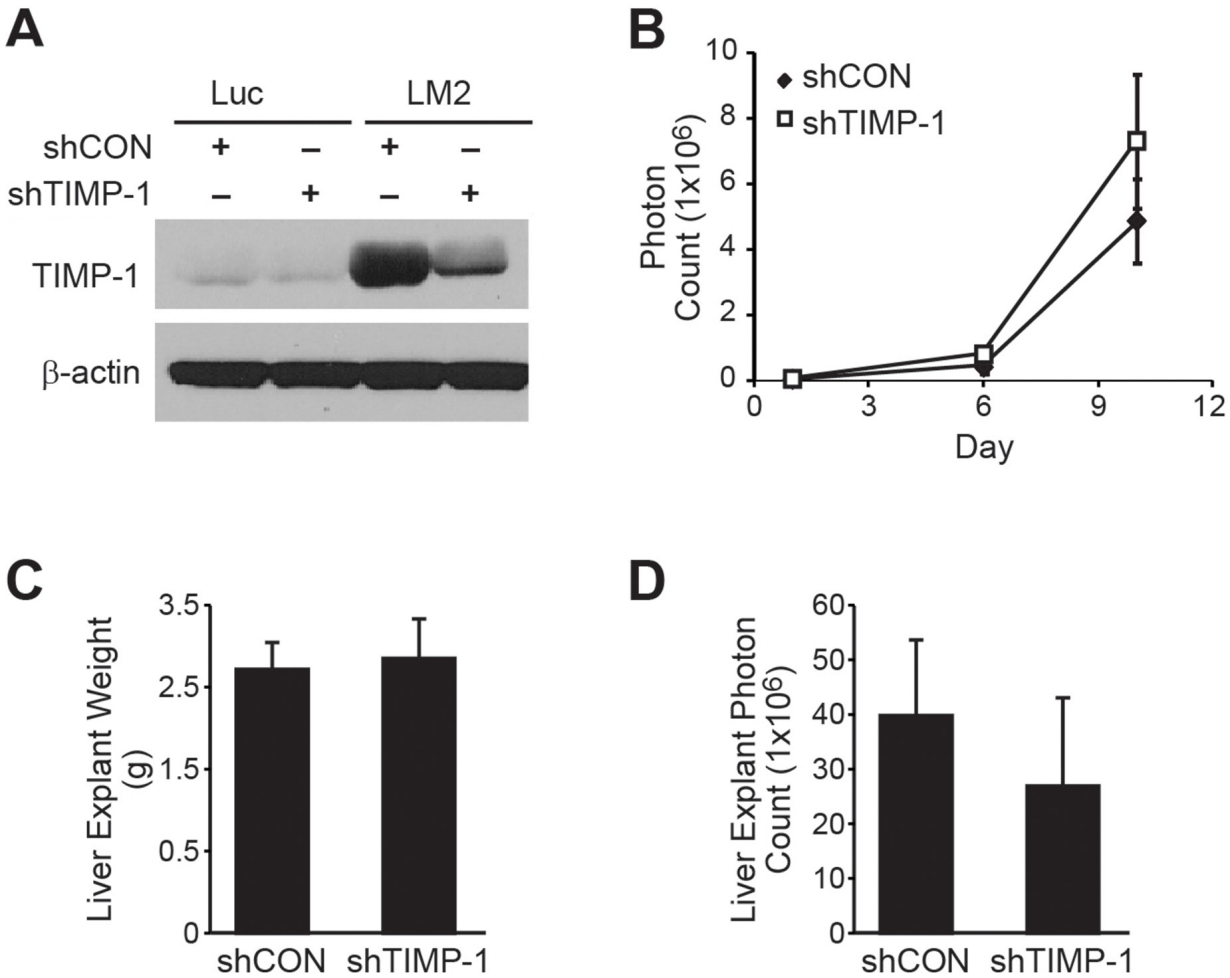
Silencing TIMP-1 fails to abrogate the prometastatic phenotype of BE(2)-C/LM2 **(A)** TIMP-1 silencing by shRNA was confirmed by immunoblotting. **(B)** No difference in *in vivo* bioluminescence was observed between BE(2)-C/LM2/shCON and BE(2)-C/LM2/shTIMP-1. **(C)** Liver explant weights (*p*= 0.95) and **(D)** bioluminescence were similar between the two groups (*p*= 0.55).

We employed our stably-transfected BE(2)C/LM2/shTIMP-1 cell line in our splenic injection model of liver metastasis to determine whether endogenous TIMP-1 expression is capable of reversing the prometastatic BE(2)-C/LM2 phenotype. The stability of our transfections was confirmed by immunoblotting prior to injection (Figure 5A). No differences were noted in serial *in vivo* bioluminescence over the two week study period (Figure 5B; *p*=0.39). Furthermore, there were no differences in liver explant mass (Figure 5C; shCON vs. shTIMP-1; 2.8g vs. 2.9g; *p*=0.95) or explant bioluminescence (Figure 5D; Con vs. shTIMP-1; 4.0x10^8^ vs. 2.7x10^8^; *p*=0.55) noted at completion of the study. These results were repeated three times with five mice per group (shCON vs. shTIMP-1). In an attempt to determine whether a difference in metastatic potential was discernible at a lower tumor cell inoculum, we attempted an additional trial by lowering the total number of injected cells by a factor of 10 (from 500,000 to 50,000 cells per splenic injection). These results successfully delayed overall liver tumor burden and time to overwhelming burden, but no differences in *in vivo* bioluminescence were noted between mice injected with BE(2)-C/LM2/shCON and BE(2)-C/LM2/shTIMP-1 cells (Supplementary Figure 4). As such, we concluded that suppression of endogenous TIMP-1 in our aggressive neuroblastoma subclone was not sufficient to rescue its prometastatic properties within our metastasis model.

### High TIMP-1 expression is associated with disease relapse and mortality in neuroblastoma

To ascertain the clinical relevance of high TIMP-1 expression in neuroblastoma, we correlated TIMP-1 expression by immunohistochemistry in our tissue microarray for advanced stage human neuroblastomas to patient outcomes. Only stages 3, 4, and 4S tumors were available in this tissue microarray. Immunohistochemical expression of TIMP-1 was scored by a pediatric pathologist (HC) blinded to the patient outcome variable. Clinical stage, *MYCN* status, disease relapse, and survival are summarized in Supplementary Table 1.

Overall, higher TIMP-1 expression was associated with adverse patient outcomes (relapse or mortality). For statistical purposes, tumors were divided into low (- or +) versus high (++ or +++) TIMP-1-expressing groups. High TIMP-1 expression was associated with increased adverse events (80% adverse event rate; 8/10) and mortality (80% mortality; 8/10), while patients with low TIMP-1 expressing tumors had fewer adverse events (30% adverse event rate; 3/10) and a more favorable prognosis (30% mortality; 3/10; Supplementary Table 1). Fisher exact test demonstrates that high TIMP-1 expression correlates with adverse events and mortality in our limited tissue microarray (p=0.04).

**Figure 6:**
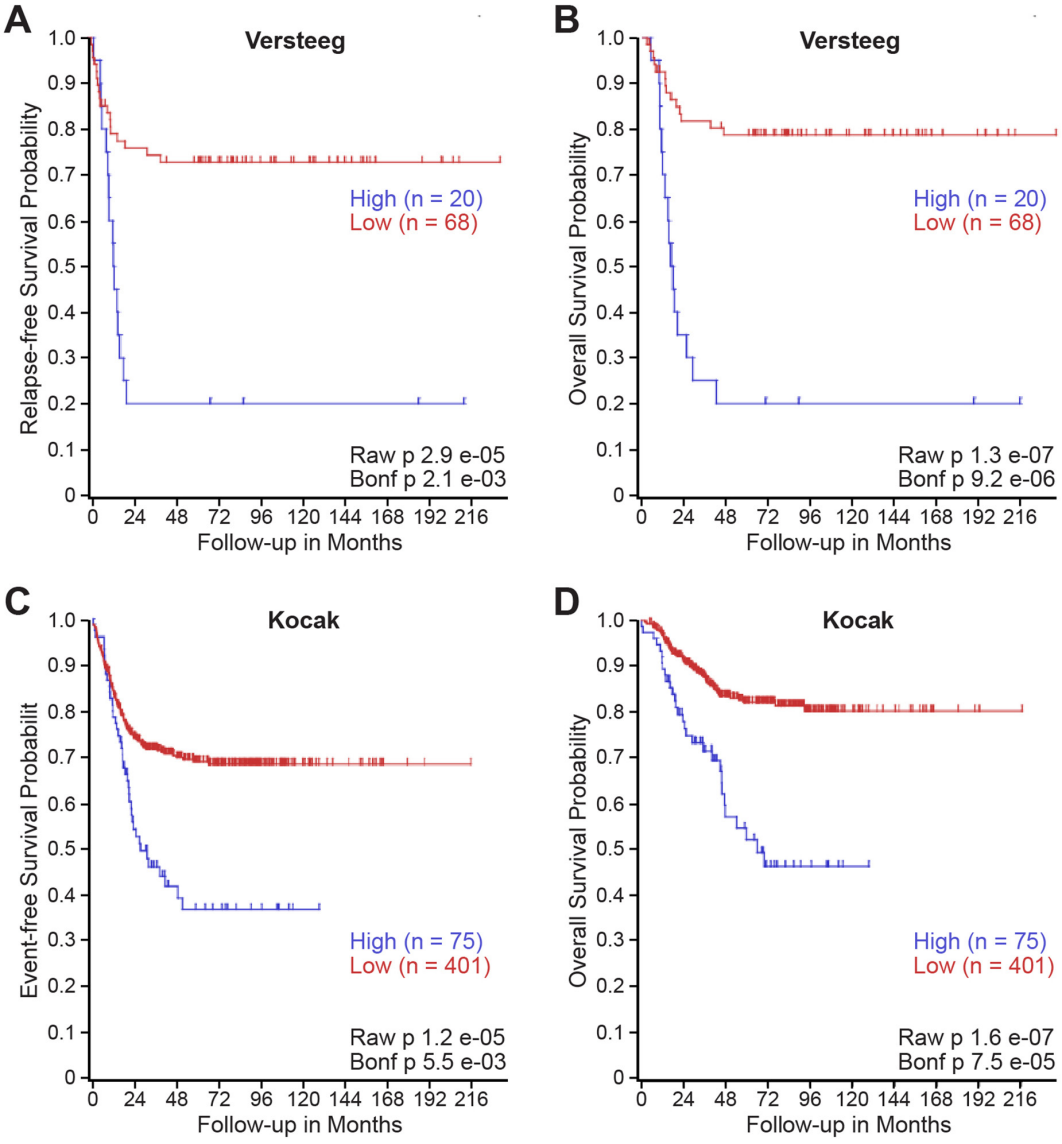
High TIMP-1 expression is associated with disease relapse and mortality in patients with neuroblastoma Kaplan–Meier curves showed the probability of **(A, C)** event-free and **(B, D)** overall survival according to levels of TIMP-1 mRNA expression in 88 and 476 human neuroblastoma samples in the publicly available microarray gene expression **(A, B)** Versteeg dataset and **(C, D)** Kocak dataset downloaded from R2 analysis and visualization platform (http://r2.amc.nl).

We utilized publically accessible clinical databases to further evaluate the relationship between TIMP-1 expression and patient prognoses in larger, independent cohorts featuring all stages of neuroblastoma. Specifically, we utilized the neuroblastoma databases of Versteeg and Kocak to generate a Kaplan-Meier Curve based upon TIMP-1 gene expression utilizing the R2: microarray analysis and visualization platform [15, 16]. As shown in Figure 6A and 6B, high TIMP-1 expression was associated with an increased risk of disease relapse (p= 2.1x10^-3^) and mortality (p= 9.2x10^-6^) within the Versteeg database. Similarly, high TIMP-1 expression was associated with recurrence (p= 5.5x10^-6^) and mortality (p= 7.5x10^-5^) within the larger Kocak database (Figure 6C and 6D). 173 samples were excluded from the Kocak database, as they lacked survival data. In Supplementary Figure 5, TIMP-1 expression was determined to be high or low using both median and high quartile level of expression as cutoffs. For further analysis, high TIMP-1 expression was associated with reduced overall survival when median (p = 0.026) and high quartile (p = 2.1x10^-6^) level of expression were used as cutoffs. High TIMP-1 expression was not associated with a survival difference in patients with *MYCN* over-amplification (Median: p = 0.616; High Quartile: p = 0.625). In *MYCN* non-amplified patients, however, high TIMP-1 expression was associated with reduced overall survival (Median: p = 0.012; High Quartile: p = 1.8x10^-5^). Together, these findings demonstrate that increased levels of TIMP-1 are associated with disease relapse and mortality in patients with neuroblastoma.

## DISCUSSION

The majority of patients with advanced stage neuroblastoma ultimately succumb to overwhelming tumor burden within distant metastatic sites [1]. In this study, we established and isolated an aggressive subclone of neuroblastoma (BE(2)-C/LM2) that reliably establishes aggressive liver metastases with decreased tumor latency and host survival (Figure 1B-1F). Intriguingly, we observed no differences in tumor burden at the site of primary implantation (the spleen) either by explant luminescence (data not shown) or weight (Figure 1D). Furthermore, implantation of BE(2)-C/LM2 cells into the subcutaneous tissue failed to result in increased tumor volume or explant mass (Supplementary Figure 1). Together, these findings suggest that BE(2)-C/LM2 is a prometastatic subclone within our intrasplenic injection model. Further validation of this prometastatic phenotype within other models of neuroblastoma metastasis, such as intracardiac injection, would inform whether our isolated subclone has a generalized propensity to establish metastases at other disease-relevant sites or if this subclone has a selective tropism for the liver [7].

The release of soluble growth factors and cytokines is critical for the establishment and survival of distant tumor metastases. As such, we utilized a cytokine array to identify host factors that may be dysregulated with implantation of our prometastatic subclone. Subsequent analysis using both mouse and human-specific ELISAs demonstrated that elevated TIMP-1 expression was present within murine host plasma and within the tumor cells themselves. TIMPs are a family of cytokines (TIMP-1, -2, -3, and -4) that are known as endogenous inhibitors of matrix metalloproteinases, enzymes involved in extracellular matrix maintenance and remodeling. In addition, TIMP-1 is also known to have an independent role in the promotion of cell division, cell growth and anti-apoptotic activity [17]. Elevated TIMP-1 expression is described as a poor prognostic plasma biomarker in several cancers including, renal cell, pancreatic, and hepatocellular carcinoma [11, 13, 14]. It is also described as a predictor of metastasis in melanoma and colon cancer [10, 12].

We chose to characterize the effects of modulating endogenous TIMP-1 expression in neuroblastoma cells utilizing RNA interference. Notably, modulating TIMP-1 expression in cell culture without potential host environmental confounders successfully abrogated adherent, soft agar colony, and tumorsphere formation within our LM2 subclone. However, stably-suppressing endogenous TIMP-1 expression failed to reverse the prometastatic phenotype of BE(2)-C/LM2 observed in our liver metastasis model. There are several potential explanations to these *in vivo* observations that may limit the generalizability of these findings. One conclusion may be that high TIMP-1 expression is a passenger characteristic of the prometastatic phenotype observed in our subclone. Another consideration is that host-derived contributions of TIMP-1 may sufficiently compensate for the effects of RNA interference within the tumor cells. Further delineation of these effects using *in vitro* co-culture with hepatocytes or establishment of a TIMP-1 deficient liver host system are considerations to further evaluate this possibility.

Similar to reports in adult cancer, high TIMP-1-expressing tumors in our small, advanced stage neuroblastoma tissue microarray (N= 20 tumors) was associated with increased adverse events and mortality (Supplementary Table 1). We broadened these observations to two larger, established neuroblastoma databases within the R2 platform and have identified elevated TIMP-1 expression to be associated with disease relapse and poor patient survival (Figure 6). To our knowledge, this is the first study associating elevated TIMP-1 with adverse outcomes in pediatric cancers. Taken together, this study demonstrated that TIMP-1 is capable of modulating neuroblastoma growth *in vitro*, and elevated expression is associated with a prometastatic phenotype in our liver metastasis model and adverse patient outcomes and mortality. These findings support further investigation into the potential role of TIMP-1 as a critical mediator of tumor-host interaction in advanced stage neuroblastoma.

## MATERIALS AND METHODS

### Materials

Antibody against TIMP-1 was purchased from Abcam (Cambridge, MA). All secondary antibodies against mouse and rabbit IgG were purchased from Santa Cruz Biotechnology, Inc. (Santa Cruz, CA). shRNA against human TIMP-1 (shTIMP-1) and non-targeting control (shCON) were purchased from Sigma-Aldrich (St. Louis, MO). Cell Counting Kit-8 (CCK-8) was purchased from Dojindo. siRNA pool against human TIMP-1 (siTIMP-1) and a scrambled sequence siRNA control (siNTC) were purchased from Santa Cruz Biotechnology.

### Cell culture, plasmids and transfection

Human neuroblastoma cell line, BE(2)-C, was purchased from ATCC (Manassas, VA) and transfected with a luciferase vector (a gift from Dr. A. Davidoff, St. Jude Hosp., Memphis, TN) to generate stable-luciferase-expressing cells (BE(2)-C/Luc). For transfection, cells were plated in 6-well plates and transfected with luciferase plasmid or shRNA (total of 4 μg) or siRNA (100 nmol) using Lipofectamine 2000™ as per manufacturer’s protocol. Zeocin (50 μg/ml) was used to select cells transfected with luciferase vector. For stable transfection of shTIMP-1 or shCON control vector, BE(2)-C/Luc cells were selected with puromycin (2.5 μg/mL).

### Liver metastasis model and *in vivo* imaging

Male athymic nude mice (4–6 weeks old) were maintained as described [18]. All studies were approved by the Institutional Animal Care and Use Committee at the Vanderbilt University Medical Center (Protocol is M1500059-00) and were conducted in accordance with guidelines issued by the National Institutes of Health. BE(2)-C/Luc, BE(2)-C/LM2, BE(2)-C/shCON, and BE(2)C/shTIMP-1 cells were used for intrasplenic injection in nude mice (n=5 for each group). Each set of experiments were completed at least three separate times. Mice were anesthetized with isoflurane and a small left flank incision was made to exteriorize the spleen. Viable cells (0.5 × 10^6^ cells/50 μl HBSS) were injected into the splenic capsule using a 27-gauge needle as described [19]. For one additional round of experiments, a lower inoculum of BE(2)-C/LM2/shCON and BE(2)-C/LM2/shTIMP-1 (0.5 × 10^5^ cells/50μL) were injected into the spleen to discern whether a difference could be noted at a lower concentration of cells.

To perform tumor imaging, mice were injected with D-luciferin (Gold Biotechnology, St. Louis, MO) subcutaneously (1 mg/mouse in 100 μl of HBSS) before being anesthetized with isoflurane. Luciferase expression of the tumors was measured using bioluminescence technology (IVIS Lumina II, Xenogen, Caliper Life Sciences, Villepinte, France) at 5 min after luciferin administration. At sacrifice, spleens and livers were isolated, weighed, and bioluminescence imaging was performed. Isolated spleens and livers were then fixed in formalin for further evaluation.

### Subcutaneous tumor formation

BE(2)-C/Luc and BE(2)-C/LM2 were implanted into athymic nude mice to assess their capacity to elicit subcutaneous xenograft formation. In brief, cells were trypsinized and resuspended in HBSS at 1x10^6^ cells/100μL. BE(2)-C/Luc and BE(2)-C/LM2 cells were injected into the subcutaneous tissue lateral to the right and left scapular region, respectively (n=5 mice per group). Xenograft volumes were measured weekly using calipers and estimated using the following formula [(length *x* width^2^)/2]. At time of sacrifice, tumor explant mass was measured.

### Anchorage-independent growth, tumorsphere assay and cell proliferation

For anchorage-independent growth assay, cells were trypsinized and resuspended in RPMI medium 1640 containing 0.4% agarose and 10% FBS. Transfected BE(2)-C cells were overlaid onto a bottom layer of solidified 0.8% agarose in RPMI medium 1640 containing 5% FBS, at cell concentrations of 3x10^3^ cells per well of a 12-well plate, and incubated for 3 weeks. Colonies were stained with 0.05% crystal violet, photographed, and quantified. For the tumorsphere assay, 1X10^3^ cells were plated on low-attachment petri dishes and grown in neural stem cell medium supplemented with B27, EGF and bFGF. Tumorspheres were imaged using a bright-field microscope and then stained with 0.05% crystal violet for quantification. To assess cellular proliferation, a modified MTT assay was used (Cell Counting Kit-8, Dojindo). The values, corresponding to the number of viable cells, were read at OD450 with the FlexStation 3 Microplate Reader (Molecular Devices, Sunnyvale, CA).

### Immunoblotting

Cells were collected using cell lysis buffer at various time points and denatured samples were prepared for immunoblotting. Target proteins were detected by using rabbit or mouse anti-human antibodies (1:500–2000 dilution) overnight at 4 °C. Anti-rabbit or anti-mouse secondary antibodies conjugated with HRP was incubated for 1 hr and visualized using an enhanced chemiluminescence system.

### Cytokine array and TIMP-1 ELISA

Mouse cytokine antibody array (cat. # ARY006) and TIMP-1 ELISA kits for human TIMP-1 (cat. # DTM100) and mouse TIMP-1 (cat. # MTM100) were purchased from R&D Systems (Minneapolis, MN). At the time of sacrifice, mouse blood was collected by cardiac puncture and stored in EDTA. Murine blood was immediately transferred into chilled tubes on ice. Blood samples were centrifuged at 2,000 × g for 10 min at 4°C. Plasma supernatant was immediately transferred into clean tubes and stored at -80°C for cytokine array assay and TIMP-1 ELISA. Cytokine antibody array analysis as well as human- or mouse-specific TIMP-1 ELISA was performed according to manufacturer’s protocol. Specificity and cross-reactivity of human- and mouse-derived TIMP-1 was tested according to the manufacturer’s protocol. Cell culture supernatant from BE(2)-C/Luc and BE(2)-C/LM2 was used to confirm the absence of cross-reactivity of human-derived TIMP-1 with mouse-derived TIMP-1. Conversely, plasma from mice injected with BE(2)-C/Luc or BE(2)-C/LM2 cells was used to confirm the lack of cross-reactivity with human-derived TIMP-1.

### Tissue microarray construction

The surgical pathology neuroblastoma specimen database at Vanderbilt University Medical Center from 1992 to 2011 was used (Vanderbilt IRB protocol #111723) [20]. A Beecher Instruments Manual Tissue Arrayer was used to prepare tissue cores from selected regions of archival tissue blocks. Four 1 mm cores were prepared for each tumor case.

### Immunohistochemistry

Tissues were fixed in formalin for 3 days and embedded in paraffin wax. Paraffin-embedded sections (5 μm) were deparaffinized in three xylene washes followed by a graded alcohol series, antigen retrieval performed with 10 mM sodium citrate buffer, and then blocked with blocking solution for 1 h at RT. Sections were incubated with primary antibody against TIMP-1 overnight at 4 °C. They were washed with phosphate buffered solution and incubated with secondary antibodies for 30 min at RT. Sections were developed with DAB reagent. Sections were counterstained with hematoxylin, dehydrated with ethanol and xylene. Coverslips were mounted and slides observed by light microscopy. Blinded scoring (0-+++) was performed by a pediatric pathologist (H.C.), where no TIMP-1 expression was scored as 0, low and medium was given + or ++ respectively, and high expression was scored +++.

### Statistical analysis

Scoring index was expressed as means ± SEM; statistical analyses were performed using student t-test for comparisons between the treatment groups. Fisher’s Exact Test was for comparison of categorical variables, such as relapse and survival for our tissue microarray studies. A p value of < 0.05 was considered significant. Kaplan-Meier survival analysis was performed using the R2: Genomics Analysis and Visualization Platform (http://r2platform.com) and log-rank test of significance was performed.

## SUPPLEMENTARY MATERIALS FIGURES AND TABLE



## Abbreviations

TIMP: tissue inhibitor of metalloproteinases
