# Supplementary material for: Elevated TIMP-1 expression is associated with a prometastatic phenotype, disease relapse, and poor survival in neuroblastoma
Source: Oncotarget. 2017 Jul 28;8(47):82609–20. doi: 10.18632/oncotarget.19664 (PMC5669914; doi:10.18632/oncotarget.19664)
Supplement: Supplementary file 1 [file oncotarget-08-82609-s001.pdf]

Elevated TIMP-1 expression is associated with a prometastatic phenotype, disease relapse, and poor survival in neuroblastoma

SUPPLEMENTARY MATERIALS

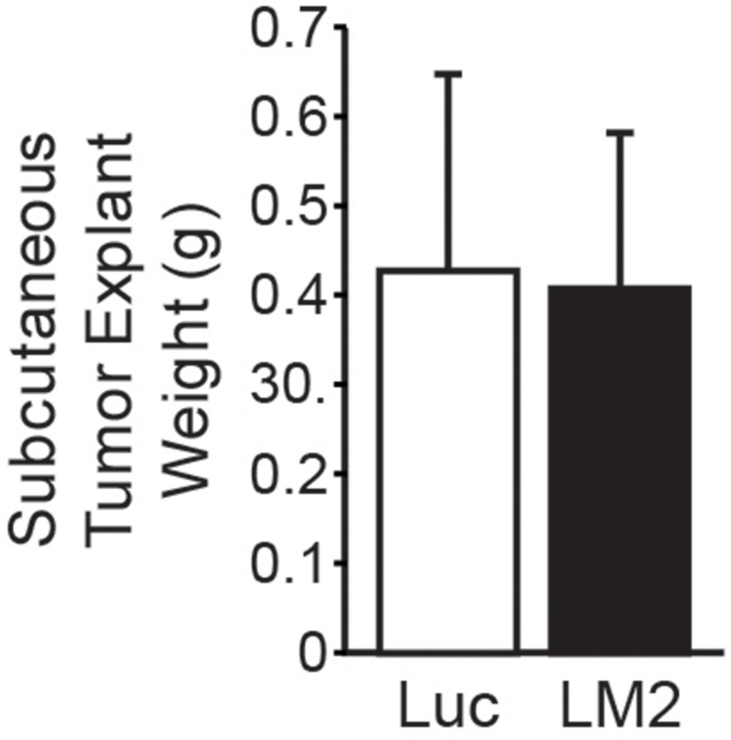

Supplementary Figure 1: BE(2)-C/LM2 subclone does not generate larger subcutaneous tumors. Parental BE(2)-C/Luc and BE(2)-C/LM2 cells were injected into the bilateral flanks of male athymic nude mice (n=5 mice). No difference in tumor explant mass was detected at the conclusion of the four week study (mean ± SEM; p= 0.73).

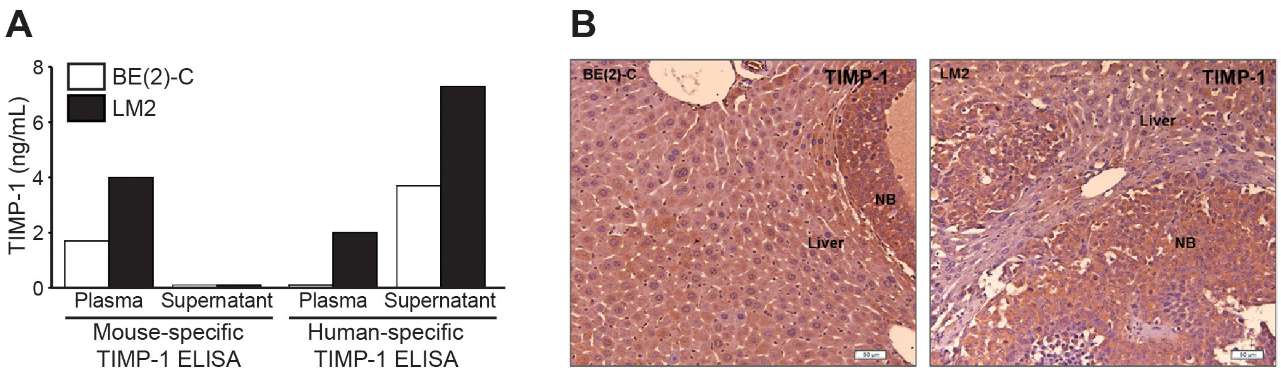

Supplementary Figure 2: Controls for *in vivo* TIMP-1 quantification. **(A)** Mouse TIMP-1 ELISA did not detect human TIMP-1 in BE(2)-C culture supernatant, but detected the TIMP-1 from plasma of mice engrafted with BE(2)-C or LM2 cells. Human TIMP-1 ELISA detected TIMP-1 from both cell culture supernatant and mice plasma. **(B)** Immunostaining for TIMP-1 in the hepatic lesions of mice injected with BE(2)-C/Luc versus BE(2)-C/LM2 with H&E to permit visualization of hepatic parenchyma and neuroblastoma tumor deposits.

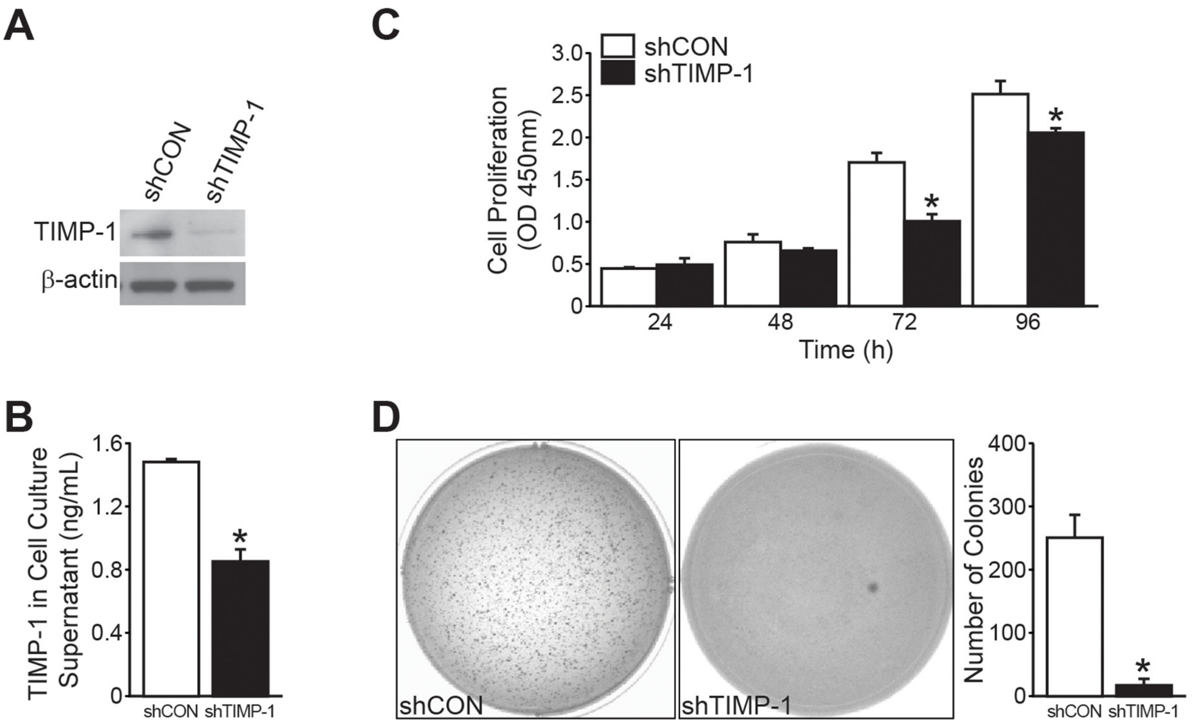

Supplementary Figure 3: shRNA-mediated TIMP-1 silencing reduced neuroblastoma growth *in vitro*. BE(2)-C/LM2 cells were transfected with shRNA against control (shCON) or TIMP-1 (shTIMP-1). **(A, B)** Immunoblotting and human-specific TIMP-1 ELISA confirmed shRNA-mediated TIMP-1 silencing, respectively. **(C)** Rate of proliferation of BE(2)-C/LM2/shTIMP-1 was markedly reduced in comparison to BE(2)-C/LM2/shCON. **(D)** Targeting TIMP-1 using shRNA inhibited anchorage-independent growth of BE(2)-C/LM2/shTIMP-1 cells in comparison to BE(2)-C/LM2/shCON cells (mean ± SEM; * =$p < 0.05$).

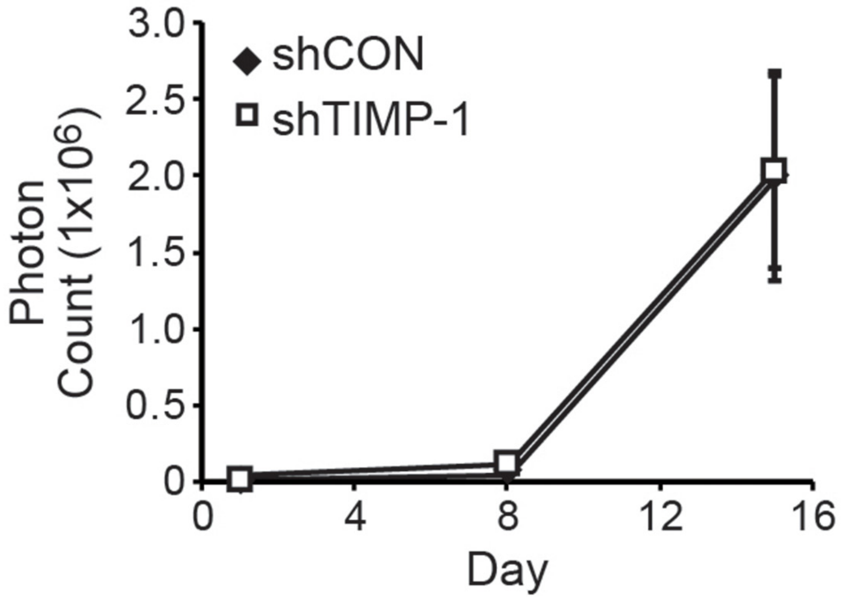

Supplementary Figure 4: Lowering splenic inoculum fails to elicit a difference in *in vivo* metastasis with TIMP-1 silencing. The splenic tumor cell inoculum was decreased to by a factor of 10 (from 500,000 to 50,000 cells) from our standard model conditions. Tumor latency was increased but no difference in *in vivo* bioluminescence was observed over the 15 day trial period (p=0.98).

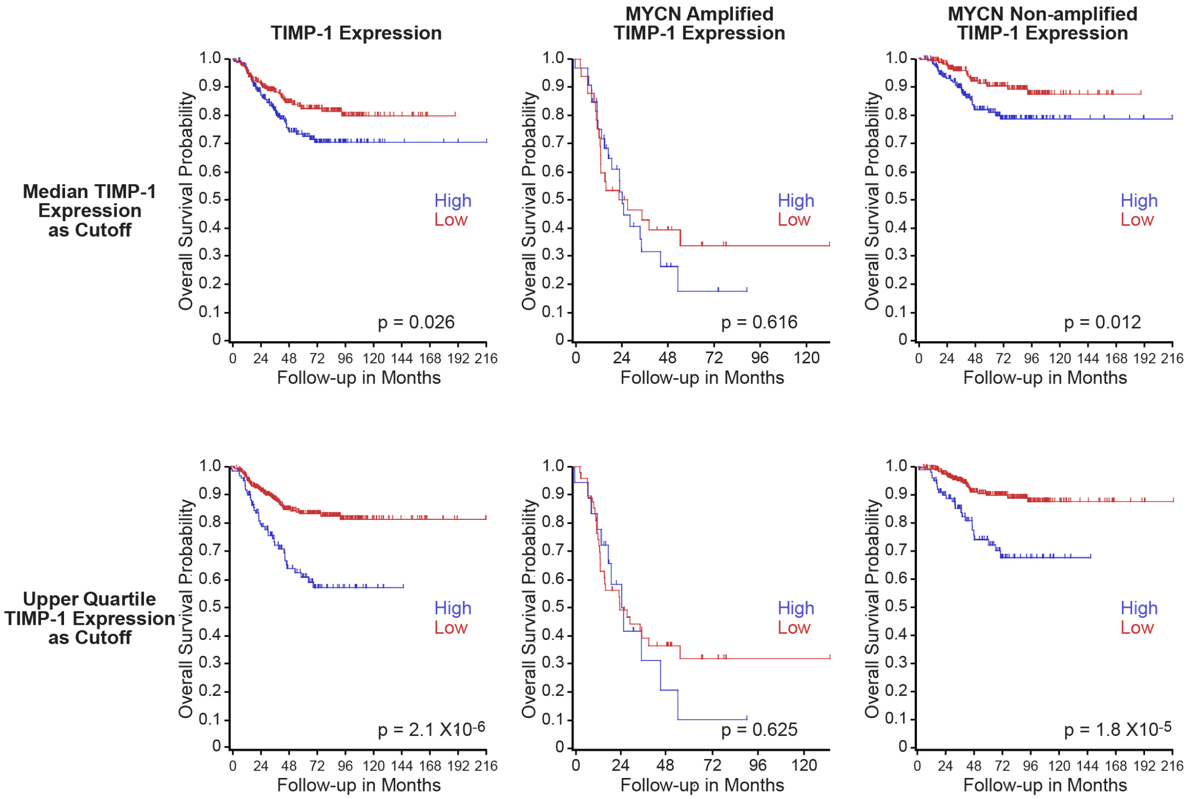

Supplementary Figure 5: Kaplan-Meier Analysis of TIMP-1 using median and high-quartile cutoffs. The Kocak microarray expression dataset was used with TIMP1 expression determined to be high or low using both median and high quartile level of expression as cutoffs [16]. High TIMP1 expression was associated with reduced overall survival when median ($p = 0.026$) and high quartile ($p = 2.1 \times 10^{-6}$) level of expression were used as cutoffs. High TIMP1 expression was not associated with a survival difference in patients with *MYCN*-amplification (Median: $p = 0.616$; High Quartile: $p = 0.625$). In *MYCN* non-amplified patients, however, high TIMP1 expression was associated with reduced overall survival (Median: $p = 0.012$; High Quartile: $p = 1.8 \times 10^{-5}$).

Supplementary Table 1: Tissue microarray demonstrates that increased TIMP-1 expression is associated with decreased survival in advanced stage (III, IV, IVs) neuroblastoma

Number	Stage	*MYCN* amp	Relapse	Status	TIMP–1
**1**	3	Yes	No	Alive	++
**2**	4S	No	No	Alive	–
**3**	4	Yes	No	Alive	–
**4**	4	No	Yes	Alive	–
**5**	4	No	No	Alive	++
**6**	4	Yes	No	Alive	–
**7**	4S	No	No	Alive	+
**8**	4S	No	No	Alive	+
**9**	4	No	No	Alive	+
**10**	4	No	Refractory	Likely deceased	++
**11**	4	Yes	Refractory	Deceased	+++
**12**	4	No	Refractory	Deceased	++
**13**	4	Yes	Refractory	Deceased	++
**14**	4	Yes	Refractory	Deceased	++
**15**	3	Yes	Yes	Deceased	+
**16**	4		Yes	Deceased	+
**17**	4S	Yes	Yes	Deceased	++
**18**	4	Yes	Yes	Deceased	+
**19**	4	No	No	Deceased	++
**20**	3	Yes	Yes	Deceased	++

Clinical variables (age, stage at presentation, *MYCN*-amplification) recurrence status, and survival are displayed in correlation with relative TIMP-1 expression quantification as determined by a blinded pediatric pathologist. High TIMP-1 expression (++ or +++) was associated with increased mortality as compared to patients with weak TIMP-1 expression (- or +; 8/10 vs. 3/10; p= 0.04).
